# Spotlight on USP4: Structure, Function, and Regulation

**DOI:** 10.3389/fcell.2021.595159

**Published:** 2021-02-18

**Authors:** Binbin Hu, Dingyue Zhang, Kejia Zhao, Yang Wang, Lijiao Pei, Qianmei Fu, Xuelei Ma

**Affiliations:** ^1^Department of Biotherapy, West China Hospital, Sichuan University, Chengdu, China; ^2^Department of Thoracic Surgery, West China Hospital, Sichuan University, Chengdu, China

**Keywords:** USP4, deubiquitinase, NF-κB, cancer, inflammation

## Abstract

The deubiquitinating enzyme (DUB)–mediated cleavage of ubiquitin plays a critical role in balancing protein synthesis and degradation. Ubiquitin-specific protease 4 (USP4), a member of the largest subfamily of cysteine protease DUBs, removes monoubiquitinated and polyubiquitinated chains from its target proteins. USP4 contains a DUSP (domain in USP)–UBL (ubiquitin-like) domain and a UBL-insert catalytic domain, sharing a common domain organization with its paralogs USP11 and USP15. USP4 plays a critical role in multiple cellular and biological processes and is tightly regulated under normal physiological conditions. When its expression or activity is aberrant, USP4 is implicated in the progression of a wide range of pathologies, especially cancers. In this review, we comprehensively summarize the current knowledge of USP4 structure, biological functions, pathological roles, and cellular regulation, highlighting the importance of exploring effective therapeutic interventions to target USP4.

## Introduction

Normal cellular homeostasis depends on the balance of protein synthesis and degradation. In eukaryotes, protein degradation is mainly carried out by the ubiquitin–proteasome system (UPS). Through a cascade between the ubiquitin-activating enzyme (E1), ubiquitin-conjugating enzyme (E2), and ubiquitin ligase (E3), the substrate proteins are decorated with ubiquitin and transferred to the 26S proteasome where they undergo degradation (Hershko et al., 1983; Hershko and Ciechanover, 1998). Ubiquitin is a 76-amino-acid protein and contains seven lysine (Lys) residues (K6, K11, K27, K29, K33, K48, and K63). These different Lys residues serve as substrates to form ubiquitin chains with distinct structures and functions. Therefore, in addition to degradation, ubiquitin modifications can also direct proteasome-independent fates, such as alteration of protein subcellular localization and activity, or involvement in histone function, DNA repair and replication, signal transduction, and other cellular processes (Hicke, 2001; Pickart and Fushman, 2004; Kulathu and Komander, 2012).

Like many other posttranslational modifications (PTMs), ubiquitination is a dynamic and reversible process. Deubiquitinating enzymes (DUBs) antagonize ubiquitination by removing ubiquitin moieties from substrate proteins (Amerik and Hochstrasser, 2004). Approximately 100 DUBs have been identified in humans, which are classified into six families consisting of the ubiquitin-specific proteases (USPs), ubiquitin C-terminal hydrolases (UCHs), ovarian-tumor proteases (OTUs), Machado–Joseph disease protein domain proteases (MJDs), JAMM/MPN domain-associated metallopeptidases (JAMMs) (Amerik and Hochstrasser, 2004), and monocyte chemotactic protein-induced proteases (MCPIPs) (Liang et al., 2010). Among them, the USP family is the largest, containing almost 60 members with each displaying diverse substrate specificity, modification preference, and regulatory mechanism.

USP4, also known as ubiquitous nuclear protein (UNP), is one of the best-studied USPs. USP4 was initially discovered, cloned, and characterized by Gupta et al. (1993) as a nuclear oncoprotein related to tre-2/tre-17/USP6. Later, it was found to contain the USP cysteine (Cys) and histidine (His) boxes and deubiquitinating activity and then proved to be USPs (Gray et al., 1995; Gilchrist et al., 1997; Frederick et al., 1998; Gilchrist and Baker, 2000). Tumor suppressor pRb is the first characterized substrate of USP4 (Blanchette et al., 2001; DeSalle et al., 2001), and later studies revealed many others. USP4 is now known to deubiquitinate many well-known target proteins associated with key processes involved in both cellular homeostasis and disease, especially cancer where USP4 is frequently dysregulated (Wang Y. et al., 2020).

This review comprehensively describes the current knowledge concerning all aspects of USP4. First, we provide an introduction to the USP4 gene and its structure, including domain architecture, catalytic activity, and substrate-binding sites. Second, we discuss the biological functions of USP4 in normal states and its pathological roles in diseases including a summary of its protein substrates. Third, we focus on the many different mechanisms involved in the cellular regulation of USP4. Finally, the developmental status of pharmacological interventions against USP4 is described. Altogether, this analysis provides fundamental insights into the potential of USP4 as a therapeutic target in multiple disease states.

## Chromosomal Location, Isoforms, and Subcellular Localization of USP4

The human USP4 gene is located on chromosome band 3p21.3 and comprised 22 exons (Gray et al., 1995). The gene encodes two dominant protein isoforms designated long and short that possess 963 and 916 amino acids, respectively (Frederick et al., 1998). The mRNA of USP4 is ubiquitously expressed in most normal tissues and cell lines at constant levels except in skeletal muscle, heart, pancreas, and testis where higher mRNA levels are detected, and in embryonic stem cells where expression is considerably lower (Gupta et al., 1994; Frederick et al., 1998). However, the subcellular localization of the USP4 protein often differs (Di Fruscio et al., 1998; Frederick et al., 1998; Soboleva et al., 2005). For example, USP4 is only expressed in the cytoplasm of HepG2 [human hepatocellular carcinoma (HCC)] cells, while being exclusively nuclear in Saos-2 (human osteosarcoma) cells (Soboleva et al., 2005). This discrepancy is partially due to nuclear–cytoplasmic shuttling where USP4 possesses both nuclear export signal and nuclear localization signal (NLS) motifs (Soboleva et al., 2005). Other mechanisms involving phosphorylation and associations with some regulators are also involved in modulating USP4 subcellular localization (Song et al., 2010; Zhang et al., 2012). Moreover, two isoforms of USP4 also have distinct subcellular distributions. While the short isoform is distributed throughout the cell, the long isoform tends to be cytoplasmic, but the regulatory mechanism remains unknown (Frederick et al., 1998). It is noteworthy that the expression ratio of the two isoforms seems to be pathological relevant as it was found to be altered explicitly in Paget disease of the bone (Klinck et al., 2014).

## Structure of USP4

**FIGURE 1:**
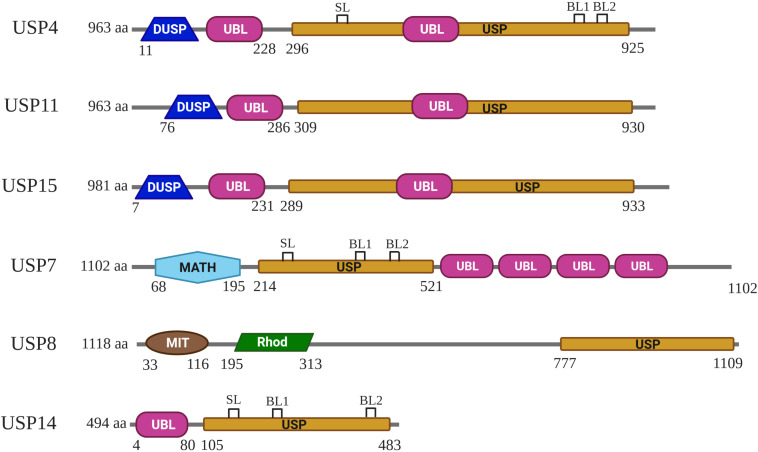
Schematic representation of the domain composition for USP4, USP11, USP15, USP7, USP8, and USP14. DUSP, UBL, USP, MATH, MIT, and Rhod domains are represented in different colors. The domain architecture of these USPs shown is based on Komander et al. (2009). SL (switching loop), BL1 (blocking loops 1), and BL2 (blocking loops 2) of USP4, USP7, and USP14 shown are based on Clerici et al. (2014), and Wertz and Murray (2019).

**FIGURE 2:**
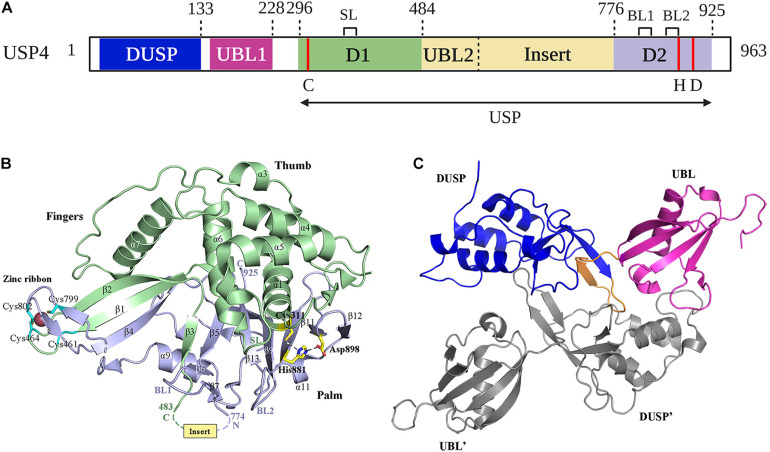
Structure of USP4. **(A)** Schematic illustration of the domain organization of USP4 in detail. The position of the catalytic residues (C, H, D) is indicated in red. SL spans residues 385–392; BL1 and BL2 span residues 831–834 and 874–880, respectively. Cartoon representation of **(B)** USP4-D1D2 domain crystal structure and **(C)** USP4 DUSP-UBL dimer structure are colored as in **(A)**. The catalytic triad and zinc-coordinating residues are represented as sticks. Dotted lines indicate the position of the USP4 insert.

Like all other USPs, USP4 is a multiple domain protein. USP4 is identified to be a paralog of USP15 and USP11, sharing a common domain organization, which consists of a DUSP (domain in USP), two UBL (ubiquitin-like), and a bi-part catalytic domain (also known as USP domain) (Angelats et al., 2003; Komander et al., 2009; Elliott et al., 2011; Vlasschaert et al., 2015) (Figure 1). UBL1 is located in USP4 N-terminal, whereas UBL2 is embedded in the C-terminal catalytic domain as a part of the insert (Ye et al., 2009; Clerici et al., 2014) (Figure 2A). Both UBL1 and UBL2 are crucial for the catalytic activity of USP4.

### C-Terminal Catalytic Domain

The catalytic core of USP4 is a bi-part domain (residues 296–925) located in C-terminal, which contains the domain 1 (D1), the domain 2 (D2), and a UBL-containing insert (Clerici et al., 2014; Vlasschaert et al., 2015) (Figure 2A). The crystal structure of USP4-D1D2 shows six molecules of USP4-D1D2 per asymmetric unit with equivalent conformation, while the insert, located between beta-strand β3 and helix α9, has an asymmetric distribution (Clerici et al., 2014). USP4–D1D2 adopts the characteristic fold seen for all USPs, which resembles an extended right hand that comprised three domains: fingers, thumb, and palm (Hu et al., 2002) (Figure 2B). The D1 fragment contains the thumb and part of the fingers, including the Cys box (residues 303–320) and QQD box (residues 390–403) of the active site, whereas the D2 fragment completes the active site with the His box (residues 864–885, 894–903, and 915–922) and the rest of the fingers and the palm (Quesada et al., 2004). Like other USPs with the exceptions of USP7 (Hu et al., 2002) and USP15 (Ward et al., 2018), the catalytic triad of USP4 is in a catalytically competent configuration (Hu et al., 2005; Avvakumov et al., 2006; Renatus et al., 2006; Komander et al., 2008; Samara et al., 2010).

USP4 and other Cys proteases have a catalytic mechanism like that of the well-studied plant enzyme papains (Storer and Ménard, 1994). First, the His residue deprotonates the thiol group of the Cys residue. Second, the Cys residue launches a nucleophilic attack on the isopeptide bond, releasing the ε-amine of the target Lys residue and producing a covalent acylenzyme intermediate with ubiquitin. Lastly, with the help of water molecules, USP4 undergoes diacylation. Consequently, free ubiquitin is released. The aspartic acid (Asp) residue is required to polarize the His residue to stabilize the enzyme’s catalytic activity (Komander et al., 2009). Several structures have a role in USP4 activation. In the fingers, the Zn^2+^ ion brings together the D1 and D2, tetrahedrally coordinated by Cys on anti–parallel β-strands β1 and β2 in D1, and β4 in D2 (Clerici et al., 2014) (Figure 2B). This zinc-finger ribbon seems to be in the contracted “closed-hand” configuration seen in USP8 that blocks ubiquitin access (Avvakumov et al., 2006) (Figure 1). A similar role was assigned to three ubiquitin-binding surface loops (BLs) observed in USP4, of which the BL1 and BL2 were reported in USP14, where they block the active site and relocate after ubiquitin binding, and the third loop corresponding to the SL plays a role in USP7 activation (Hu et al., 2002; Faesen et al., 2011) (Figures 1, 2A,B). Superposition of the six non-crystallographic symmetry-related molecules of USP4–D1D2 shows that both the zinc-finger ribbon and the three BLs have flexibility, which is required for the dynamic exchange of ubiquitin in and out of the active site (Clerici et al., 2014).

### N-Terminal DUSP-UBL Domain

The N-terminal DUSP-UBL domain spans residues 1–228, separated from the catalytic domain by a connecting linker (residues 229–295) (Clerici et al., 2014) (Figure 2A). DUSP-UBL domain exists not only in USP4 but also in USP15 and USP11 (Elliott et al., 2011) (Figure 1). The crystal structure of USP4 DUSP-UBL shows a dimer formation where the subunits are packed against each other in an antiparallel orientation (Harper et al., 2011) (Figure 2C). This packing creates an extensive interface formed through the DUSP domains, but not UBL domains from the different subunits, suggesting that the N-terminal UBL domain has an independent motion (Elliott et al., 2011).

The primary function of the DUSP-UBL domain is to activate USP4 by promoting catalytic domain turnover. A study conducted by Clerici et al. (2014) revealed that the DUSP-UBL domain could not only improve the ubiquitin off-rates but also promote faster on-rates through directly binding to the insert. In addition, deletion of the DUSP, the insert, or the linker region that separates the DUSP-UBL to the catalytic domain from USP4 showed that losing only one of these three domains can reduce the catalytic activity of USP4. Thus, the DUSP-UBL, the linker, and the inserted domain are essential for USP4 activation. Two isoforms of USP4 arise from the inclusion or skipping of exon 7 (E7), which is serine-rich with several phosphorylation sites and forms the major part of the flexible linker region (Vlasschaert et al., 2016). Given that there are no differences in USP4 deubiquitinating activity between long and short forms, the part of the linker region formed by E7 may not be crucial for USP4 activation because the short linker retains sufficient flexibility to enable comparable domain interactions (Clerici et al., 2014; Vlasschaert et al., 2016).

### Substrate-Binding Domains

All USP4 subdomains have been found to play a role in substrate recognition (Table 1). The majority of substrates bind to the C-terminal catalytic domain, and few interact with the N-terminal DUSP-UBL domain. In addition, the binding sites may contain part of two subdomains or even three or more subdomains. For example, histone deacetylase 2 (HDAC2) associates with amino acids 188–302 of USP4 (Li Z. et al., 2016), ARF-binding protein 1 (ARF-BP1) interacts with residue 188 to C-terminus of the enzyme (Zhang et al., 2011), and RNA-binding protein with serine-rich domain 1 (RNPS1) binds to the full-length USP4 composed of DUSP, UBL1, UBL2, and UCH domains (Kwon et al., 2017).

**TABLE 1:** Substrate-binding domains in USP4.

Binding domains	Substrates	References	Binding domains	Substrates	References
The catalytic (USP) domain	TβRI	Zhang et al., 2012	DUSP domain	RIG-I	Wang et al., 2013
	CtIP	Liu et al., 2015; Wijnhoven et al., 2015		Rheb	Deng et al., 2019
	RORγt	Yang et al., 2015	UBL1 domain	S9	Zhao et al., 2012
	IRF8	Lin et al., 2017	DUSP-UBL domain	BRAP	Hayes et al., 2012
	β-Catenin	Yun et al., 2015	Amino acids 188–302	HDAC2	Li Z. et al., 2016
	TAK1	Fan et al., 2011	Residue 188 to C-terminus	ARF-BP1	Zhang et al., 2011
	TRAF2	Xiao et al., 2012	Amino acids 613–923	TAK1	Zhao et al., 2018
	TRAF6	Xiao et al., 2012; Xu et al., 2018	DUSP, UBL1, UBL2, and UCH domains	RNPS1	Kwon et al., 2017
	RIP1	Hou et al., 2013			

## Cellular and Biological Function of USP4

USP4 functions to remove monoubiquitination and polyubiquitination, including K48 and K63 conjugated ubiquitin chains, from target proteins (Zhao et al., 2009; Zhou et al., 2012). Given that numerous substrates are influenced by USP4, USP4 has been found to play an indispensable role in a wide range of cellular and biological processes, as discussed below and summarized in Table 2.

**TABLE 2:** Substrates of USP4 in cellular and biological processes.

Substrates	Type of ubiquitination removed by USP4	References	Substrates	Type of ubiquitination removed by USP4	References
Pre-mRNA splicing			Wnt/β-catenin signaling pathway		
PRP3	K63-linked ubiquitin chains	Song et al., 2010	β-Catenin	K48-linked ubiquitin chains	Yun et al., 2015
RNP1	K63-linked ubiquitin chains	Kwon et al., 2017	NLK	None reported	Zhao et al., 2009
DNA repair			TCF4	None reported	Zhao et al., 2009
CtIP	Non-deubiquitination	Liu et al., 2015; Wijnhoven et al., 2015	NF-κB signaling pathway		
Cell growth and differentiation			TRAF2	K63-linked ubiquitin chains	Xiao et al., 2012
Dvl	K63-linked ubiquitin chains	Zhou et al., 2016	TRAF6	K48- and K63-linked ubiquitin chains	Xiao et al., 2012; Zhou et al., 2012; Xu et al., 2018
MyoD	None reported	Yun and Kim, 2017	TAK1	K63-linked ubiquitin chains	Fan et al., 2011; Liang et al., 2013
SMAD4	Monoubiquitination	Zhou et al., 2017	RIP1	K63-linked ubiquitin chains	Hou et al., 2013
Immune response			RIG-I	K48-linked ubiquitin chains	Wang et al., 2013
RORγt	K48-linked ubiquitin chains	Yang et al., 2015	Hyaluronan synthesis		
IRF4	K48- and K63-linked ubiquitin chains	Guo et al., 2017	HAS2	Monoubiquitination	Mehić et al., 2017
IRF8	K48-linked ubiquitin chains	Lin et al., 2017	Other biological functions		
P53 signaling pathway			AQP2	None reported	Murali et al., 2019
HDAC2	None reported	Li Z. et al., 2016	A2a receptor	None reported	Milojevic et al., 2006
ARF-BP1	None reported	Zhang et al., 2011	BRAP	None reported	Uras et al., 2012
TGF-β signaling pathway			PDK1	Monoubiquitination	Hayes et al., 2012
TβRI	None reported	Zhang et al., 2012	S9	Non-deubiquitination	Zhao et al., 2012

### Pre-mRNA Splicing

The activity and composition of the spliceosome, which is critical for pre-mRNA splicing, are regulated by reversible ubiquitination (Nottrott et al., 2002; Jurica and Moore, 2003; Maeder and Guthrie, 2008). Song et al. (2010) showed that USP4 could deubiquitinate K63-linked polyubiquitin chains of pre-mRNA-processing factor 3 (PRP3), a component of the U4 snRNP, to promote ejection of U4 proteins from the spliceosome, thereby ensuring the correct pre-mRNA splicing. Moreover, loss of USP4 disturbs efficient pre-mRNA splicing of several cell cycle regulators, such as α-tubulin and budding uninhibited by benzimidazoles 1(BUB1), resulting in the impairment of mitotic progression (Song et al., 2010). USP4 was also reported to deubiquitinate RNPS1, which aids in selecting splicing sites and activating the pre-mRNA splicing process (Kwon et al., 2017).

### DNA Repair

USP4 has been shown to participate in DNA double-strand break resection and homologous recombination (HR) repair upon DNA damage. By directly interacting with CtIP, a DNA-end resection factor controlling the DNA damage-induced G2/M checkpoint, USP4 promotes CtIP foci formation at DNA damage sites. Although USP4 does not directly deubiquitinate CtIP, its interaction with CtIP is essential for HR as blocking their binding reduces HR efficiency (Liu et al., 2015; Wijnhoven et al., 2015).

### Cell Growth and Differentiation

Anckar and Bonni (2015) initially reported that USP4 had opposing effects on cell morphogenesis in the mammalian cerebellum where it promoted axon growth in granule neurons but concomitantly restricted the growth and elaboration of dendrites. Later studies confirmed roles for USP4 in cell growth and differentiation (Zhou et al., 2016, 2017; Yun and Kim, 2017). USP4 suppresses Wnt3a-induced osteoblast differentiation and mineralization by cleaving K63-linked polyubiquitin chains from disheveled (Dvl) and inhibiting the accumulation of β-catenin and activation of its downstream cascades (Zhou et al., 2016). Through downregulating myoblast determination protein (MyoD) activity, USP4 also exerts an antagonistic function in myoblast differentiation (Yun and Kim, 2017). Recently, SMAD4, an intracellular effector for transforming growth factor β (TGF-β) family cytokines, was shown to be a novel substrate of USP4. The binding of USP4 to SMAD4 prevents its monoubiquitination in the cytoplasm and subsequently enhances SMAD4-mediated activin and bone morphogenetic protein (BMP) pathways. In mice, this leads to the suppression of embryonic stem cell differentiation to sustain their self-renewal potential (Zhou et al., 2017), whereas, in early zebrafish embryos, this process can maintain morphogenic events (Tse et al., 2009; Zhou et al., 2017). However, a single knockout of either USP4 or USP15 does not impair viability and normal development in mice, whereas a double knockout of USP4 and USP15 leads to embryonic lethality (Vlasschaert et al., 2015). These results suggest for embryonic development that USP4 and USP15 are functionally redundant.

### Immune Response

Conflicting functions have been reported where USP4 can act as an enhancer or an inhibitor of immune responses. For example, by stabilizing retinoic acid receptor–related orphan receptor γt (RORγt), USP4 facilitates interleukin-17A (IL-17A) transcription and T helper type 17 (T_H_17) cell differentiation (Yang et al., 2015). USP4 also promotes the development of T_H_2 cells and upregulation of T_H_2-related cytokines through physically interacting with and deubiquitinating interferon regulatory factor 4 (IRF4) (Guo et al., 2017). In contrast to these activation responses, IRF8, another immune system-restricted IRF required for T-helper cell differentiation, has been recognized as a substrate of USP4. Deconjugation of polyubiquitin chains from IRF8 mediated by USP4 is vital for maintaining the function of regulatory T cells, which negatively regulate many different immune reactions (Lin et al., 2017).

### P53, TGF-β, Wnt/β-Catenin, and Nuclear Factor κB Signaling Pathways

**FIGURE 3:**
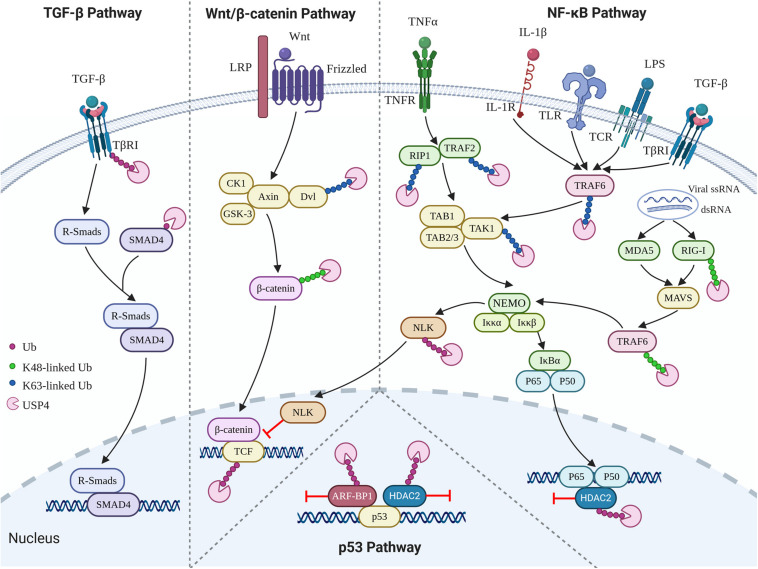
Role of USP4 in p53, TGF-β, Wnt/β-catenin, and NF-κB signaling pathways.

In addition to the biological contexts mentioned above, USP4 is also involved in the regulation of p53, TGF-β, Wnt/β-catenin, and nuclear factor κB (NF-κB) signaling pathways (Figure 3). USP4 inhibits p53 transcriptional activation via stabilizing HDAC2 and ARF-BP1, which acts as a deacetylase and E3 ubiquitin ligase of p53, respectively (Zhang et al., 2011; Li Z. et al., 2016). Loss of UPS4 leads to the activation of several p53-directed pathways, including upregulation of apoptosis, premature cell senescence, and reduced oncogene-associated transformation (Zhang et al., 2011). USP4 can also deubiquitinate TGF-β type I receptor (TβRI) and sustain its plasma membrane expression in a SMAD7-independent fashion, leading to hyperactivation of the TGF-β pathway (Zhang et al., 2012). The Wnt/β-catenin signaling pathway is an important pathway governing developmental, homeostatic, and pathological processes (Clevers and Nusse, 2012). USP4 was initially identified as a repressor of the Wnt/β-catenin signaling pathway, given that USP4 can mediate deubiquitination and stabilization of Dvl, a key molecule involved in the turnover of cytosolic β-catenin (Zhao et al., 2009; Zhou et al., 2016). However, later studies demonstrated that USP4 enhanced Wnt signaling through stabilizing and facilitating nuclear localization of β-catenin, especially in cancer (Yun et al., 2015; Hwang et al., 2016). Furthermore, two other Wnt signaling components, transcription factor 4 (TCF4) and Nemo-like kinase (NLK) have also been identified as substrates of USP4 (Zhao et al., 2009).

NF-κB, a transcription factor involved in a broad range of cellular responses such as inflammation, immunity, and apoptosis, has been extensively reported to be negatively regulated by USP4 (Fan et al., 2011; Xiao et al., 2012; Zhou et al., 2012; Hou et al., 2013; Wang et al., 2013; Li Z. et al., 2016; Wang X. D. et al., 2020). Tumor necrosis factor receptor–associated factor 2 (TRAF2), TRAF6, and TGF-β activated kinase 1 (TAK1) are three crucial NF-κB signaling components. K63-linked polyubiquitination of TAK1 mediated by K63-linked polyubiquitination of TRAF2 or TRAF6 is an essential step in IκB phosphorylation, degradation, and further translocation of NF-κB to the nucleus and upregulation of the subsequent proinflammatory responses (Deng et al., 2000; Wu et al., 2005; Yamazaki et al., 2009; Fan et al., 2010). Several studies have indicated that USP4 acts as a deubiquitinase for TRAF2, TRAF6, and TAK1 to prevent tumor necrosis factor α (TNF-α), IL-1β-, TGF-β-, T-cell receptor (TCR)-, and lipopolysaccharide (LPS)-induced NF-κB activation (Fan et al., 2011; Xiao et al., 2012; Zhou et al., 2012; Wang X. D. et al., 2020). Besides, the suppressing role of USP4 in NF-κB signaling is also seen in its deubiquitinating activity toward receptor-interacting protein 1 (RIP1), whose K63-linked polyubiquitin chains function as scaffolds for the assembly of TAK1 kinase, and also HDAC2, which is a deacetylase associating with HDAC1 to target the p65 (RelA) subunit (Hou et al., 2013; Li Z. et al., 2016). Retinoic acid–inducible gene I–like receptors (RLRs), including retinoic acid–inducible gene I (RIG-I), are other inducible factors of the NF-κB signaling pathway (Gürtler and Bowie, 2013). Wang et al. (2013) discovered that USP4 interferes with the RIG-I-mediated activation of NF-κB. However, Xu et al. (2018) demonstrated that USP4 could positively regulate the RLR-induced NF-κB signaling pathway by removing K48-linked ubiquitin chains from TRAF6. Therefore, deubiquitinating K63- or K48-linked chains in TRAF6, respectively, may trigger anti-inflammatory or proinflammatory responses.

### Other Biological Functions

USP4 is highly expressed in the kidney where it functions to regulate whole-body water homeostasis through deubiquitinating arginine vasopressin-induced membrane accumulation of aquaporin 2 (AQP2), the water channel of the kidney collecting duct (Murali et al., 2019). USP4 also influences the expression of the G_s_-coupled receptor A_2__a_ adenosine receptor in cultured hippocampal neurons, acting to relax its quality control in the endoplasmic reticulum and enhancing its cell surface expression (Milojevic et al., 2006). Moreover, other studies have described BRCA1-associated protein, phosphoinositide-dependent kinase 1, and hyaluronan synthase 2 (HAS2) as substrates of USP4 (Hayes et al., 2012; Uras et al., 2012; Mehić et al., 2017). For the latter, USP4 removes monoubiquitination from HAS2, which leads to decreased hyaluronan synthesis (Mehić et al., 2017). USP4 may also take part in proteasome assembly via binding to the S9 subunit of the proteasome, although its catalytic activity is not necessary for this process (Zhao et al., 2012).

## Dysregulation of USP4 in Disease

USP4 is essential for normal physiological functions, although its dysregulation can lead to different pathologies. Frederick et al. (1998) obtained the first evidence of the link between USP4 and cancer when they demonstrated the lower expression of USP4 in small cell lung cancer and squamous cell lung cancer compared with normal samples. Since then, numerous studies have documented USP4 to function as an oncoprotein or a tumor repressor in a range of different cancer types. USP4 was also reported to have impacts on many other pathological contexts, such as viral and bacterial infections, acute injury and inflammation, chronic fibrosis-related diseases, metabolic disorders, and immune disease (Table 3). Together, these findings implicate USP4 as a suitable candidate to be targeted in a wide variety of diseases.

**TABLE 3:** Multifaceted role of USP4 in diseases.

Diseases		Substrates	Substrate-related pathways	Expression of USP4 (cells or tissues)	Role of USP4	References
Cancer	Breast cancer	TβRI	TGF-β pathway	Upregulated (human cells and clinical samples)	Promoter	Zhang et al., 2012; Cao et al., 2016
		HDAC2	Several cell proliferation-associated genes expression	None reported	Promoter	Wang et al., 2018
		PCD4	None reported	Downregulated (clinical samples)	Suppressor	Li Y. et al., 2016
	Colorectal cancer	β-Catenin	Wnt/β-catenin pathway	Upregulated (clinical samples)	Promoter	Yun et al., 2015
		Rheb	mTORC1 pathway	None reported	Promoter	Deng et al., 2019
		PRL-3	AKT pathway	Upregulated (clinical samples)	Promoter	Xing et al., 2016
	Brain metastatic lung adenocarcinoma	β-Catenin	Wnt/β-catenin pathway	Upregulated (human cells)	Promoter	Hwang et al., 2016
	Lung cancer	TRAF2, TRAF6	NF-κB pathway	None reported	Suppressor	Xiao et al., 2012
		Twist1	Oct4/Sox2 expression	Upregulated (clinical samples)	Promoter	Li et al., 2020
		None reported	NF-κB pathway	Downregulated (stemness-enriched human lung cancer cells and advanced lung cancer clinical samples)	Suppressor	Lai et al., 2020
	HNSCC	RIP1	NF-κB pathway	Upregulated (clinical samples)	Suppressor	Hou et al., 2013
	GBM	TβRI	TGF-β/ERK pathway	Upregulated (clinical samples)	Promoter	Zhou Y. et al., 2019
		None reported	P53 pathway	Upregulated (human cells and clinical samples)	Promoter	Qin et al., 2019
	Melanoma	None reported	P53 pathway	Upregulated (human cells and clinical samples)	Promoter	Guo et al., 2018
	HCC	CypA	MAPK pathway	Upregulated (clinical samples)	Promoter	Li et al., 2018
		TβRI	TGF-β pathway	Upregulated (mesenchymal-phenotype human HCC cells and clinical samples)	Promoter	Heo et al., 2014; Qiu et al., 2018
Viral infections	VSV	RIG-I	IFN-β pathway	Downregulated (virus-infected human cells)	Suppressor	Wang et al., 2013
	EV71	TRAF6	NF-κB pathway	Downregulated (virus-infected human cells)	Suppressor	Xu et al., 2018
Acute injury and inflammation	Spinal cord injury	TRAF6	NF-κB pathway	Downregulated (rat microglial cells)	Suppressor	Jiang et al., 2017
	Intracerebral hemorrhage	None reported	Caspase-3/Bax/Bcl-2 pathway	Upregulated (rat neurons adjacent to the hematoma)	Promoter	Liu et al., 2017
	Hepatic (I/R) injury	TAK1	TAK1/JNK1/2/p38 pathway	Upregulated (mouse liver tissues suffering I/R injury)	Suppressor	Zhou J. et al., 2019
Chronic fibrosis-related diseases	Cardiac hypertrophy and fibrosis	TAK1	TAK1/JNK1/2/p38 pathway	Downregulated (clinical samples)	Suppressor	He et al., 2016
	Liver fibrosis and cirrhosis	TβRI	TGF-β pathway	Upregulated (mouse fibrous liver tissues)	Promoter	Wu et al., 2019; Zhu et al., 2019
	Pathological scarring	TβRI	TGF-β pathway	Upregulated (clinical samples)	Promoter	Zhang J. et al., 2019; Huang et al., 2020
	MMT during peritoneal dialysis	TβRI	TGF-β pathway	None reported	Promoter	Xiao et al., 2015
Metabolic disorders	NAFLD	TAK1	TAK1/JNK/IRS/AKT/GSK3 pathway	Downregulated (clinical samples)	Suppressor	Zhao et al., 2018; Zhang Y. et al., 2019
Immune disease	RHD	RORγt	IL-17A transcription	Upregulated (clinical samples)	Promoter	Yang et al., 2015
		IRF4	IL-4 transcription	None reported	Promoter	Guo et al., 2017

### Cancer

Examining 14 types of human cancer, Zhang et al. (2011) reported that the expression level of USP4 was upregulated in the majority of cases. A series of tumors, including breast cancer, colorectal cancer, lung cancer, brain metastatic lung adenocarcinoma, glioblastoma (GBM), melanoma, HCC, and adrenocortical carcinoma, overexpress USP4. Moreover, USP4 overexpression contributes to the initiation, progression, and/or the recurrence of tumors and is correlated with poor prognosis (Velázquez-Fernández et al., 2005; Laurell et al., 2009; Zhang et al., 2012; Yun et al., 2015; Cao et al., 2016; Hwang et al., 2016; Xing et al., 2016; Guo et al., 2018; Li et al., 2018, 2020; Qiu et al., 2018; Wang et al., 2018; Deng et al., 2019; Qin et al., 2019; Zhou Y. et al., 2019; Geng et al., 2020). In breast cancer, higher expression of USP4 is associated with distant metastasis (Geng et al., 2020). By promoting activation of the relaxin/TGF-β1/Smad2/matrix metalloproteinase 9 axis and transcriptional repression activity of HDAC2, USP4 induces breast cell invasion, migration, and proliferation both *in vitro* and *in vivo* (Cao et al., 2016; Wang et al., 2018). Phosphatase of regenerating liver-3 (PRL-3), a member of the protein tyrosine phosphatase family, plays a significant role in tumorigenesis (Liang et al., 2007; Al-Aidaroos and Zeng, 2010). Deubiquitinating PRL-3 by USP4 leads to AKT activation and E-cadherin reduction in colorectal cancer, where an elevated level of USP4 is associated with tumor size, differentiation, distant metastasis, and poor survival (Xing et al., 2016). This procancer effect is also seen in the USP4-induced deubiquitination of Rheb, which can promote mTOR complex 1–mediated cellular activities, including the control of cellular autophagy, proliferation, and cell size (Deng et al., 2019). As mentioned previously, β-catenin is a substrate of USP4, and this interaction affects the tumorigenicity of colorectal cancer, as well as brain metastasis in lung adenocarcinoma (Yun et al., 2015; Hwang et al., 2016).

Recently, USP4 was found to mediate deubiquitination of the epithelial–mesenchymal transition (EMT) transcriptional factor Twist1 to promote lung cancer cell stemness (Li et al., 2020). Zhou Y. et al. (2019) also demonstrated that inhibiting USP4 expression in GBM cells both inhibited their proliferation and induced apoptosis via antagonizing USP4-mediated stimulation of the ERK signaling pathway. Similarly, knockdown of USP4 significantly decreases cell viability in a p53-dependent manner after treating GMB cells with temozolomide (Qin et al., 2019). A comparable role was observed in melanoma where USP4 expression aggravated cisplatin chemoresistance (Guo et al., 2018). Cyclophilin A (CypA) and TβRI stabilization by USP4 occurs in HCC. Enhanced tumor growth mediated by USP4 overexpression and its correlation with several malignant phenotype characteristics provides the fundamental and clinical evidence that USP4 functions as a tumor promoter in HCC (Heo et al., 2014; Li et al., 2018; Qiu et al., 2018).

On the other hand, quite a few studies discovered that USP4 exerts suppressive functions in particular types of cancer (Xiao et al., 2012; Hou et al., 2013; Liang et al., 2013, 2019; Li Y. et al., 2016; Yao et al., 2017; Zhong et al., 2018; Lai et al., 2020). In esophageal cancer, high expression of USP4 is associated with the improved prognosis of patients presenting with small tumors or diagnosed at an early stage (Yao et al., 2017). An association between USP4 and RIP1 was reported to impair NF-κB activation and exacerbate TNF-α–induced apoptosis in head and neck squamous cell carcinoma (HNSCC) (Hou et al., 2013). Activation of NF-κB is also one of the resistance mechanisms contributing to resistance to chemotherapy and ionizing radiation during cancer treatment (Li and Sethi, 2010). Liang et al. (2013) showed that USP4 could block Dox-induced activation of NF-κB at the early stage of the treatment by preventing TAK1 from conjugating K63-linked polyubiquitin chains.

The reduced expression of USP4 in lung adenocarcinoma and lung squamous cell cancer compared with normal samples is associated with poor survival among lung cancer patients (Zhong et al., 2018). The connection between USP4 and NF-κB is identified in lung cancers where stable knockdown of USP4 augments inflammatory responses, stemness properties, chemoresistance, the expression of programmed death ligand 1, and tumor growth (Lai et al., 2020). This inhibiting effect mediated by USP4 in lung cancer is in accordance with the results from Xiao et al. (2012), while in contrast to another study reported USP4 promotes lung cancer cell stemness (Li et al., 2020). Additionally, two studies indicated that USP4 downregulation might also mediate a tumor-suppressing role in breast cancer (Li Y. et al., 2016; Liang et al., 2019). USP4 was found to interact with and stabilize programmed cell death 4 (PCD4) that is involved in the inhibition of breast cancer cell proliferation (Li Y. et al., 2016). Moreover, knockdown of USP4 facilitated the proliferation, migration, and invasion of breast cancer cells, as well as tamoxifen resistance (Liang et al., 2019). Therefore, abnormal expression of USP4 can either increase or decrease tumorigenesis depending on the cell context, suggesting a multidimensional role for USP4 in cancer.

### Viral and Bacterial Infections

Aberrant regulation of USP4 has also been reported in viral and bacterial infections. USP4 is downregulated in cells and mice infected by enterovirus 71 (EV71), a single-stranded positive-sense RNA virus of the Picornaviridae family. Overexpression of USP4 inhibits EV71 replication by stabilizing TRAF6 to activate NF-κB inflammatory signaling (Xu et al., 2018). Similarly, in vesicular stomatitis virus (VSV) infection, removing K48-linked polyubiquitin chains from RIG-I allows USP4 to enhance RIG-I-triggered IFN-β signaling, thereby inducing an antiviral immune response (Wang et al., 2013). Reductions in USP4 levels also occur in macrophages infected with *Salmonella typhimurium*, but the detailed mechanism has not been established to date (Kummari et al., 2015).

### Acute Injury and Inflammation

USP4 is expressed in microglia, but its level is reduced after spinal cord injury. Here the downregulation of USP4 promotes microglial activation and subsequent neuronal inflammation through attenuating the deubiquitination of K63-linked TRAF6 and reversing its suppressor role in the NF-κB signaling pathway (Jiang et al., 2017). In intracerebral hemorrhage, another acute form of neurological damage, USP4 regulates the caspase-3/Bax/Bcl-2 axis to enhance apoptosis in neurons (Liu et al., 2017). In contrast, USP4 exhibits a protective role in hepatic ischemia/reperfusion (I/R) injury. Using a mouse model, Zhou J. et al. (2019) showed that USP4 levels were notably elevated in livers subjected to I/R injury, which functioned to alleviate hepatocyte apoptosis and liver inflammation through USP4-induced deubiquitination of TAK1 and inactivation of TAK1/JNK1/2/p38 signaling pathway.

### Chronic Fibrosis-Related Diseases

USP4 is also involved in chronic fibrosis-related diseases. USP4 was reported to have a lower expression in the failing human heart compared with normal heart. Overexpression of USP4 can prevent angiotensin II–induced cardiac hypertrophy and fibrosis *in vitro* and ameliorate cardiac dysfunction *in vivo* through downregulation of the TAK1/JNK1/2/p38 signaling pathway (He et al., 2016). Deubiquitinating TβRI and stimulation of TGF-β signaling pathway also associate USP4 with liver fibrosis and cirrhosis where it facilitates activation of hepatic stellate cell and EMT in hepatocytes (Wu et al., 2019; Zhu et al., 2019). USP4 can promote pathological scarring in response to trauma in the skin and mesothelial-to-mesenchymal transition (MMT) during peritoneal dialysis through maintaining TβRI stability (Xiao et al., 2015; Zhang J. et al., 2019; Huang et al., 2020).

### Metabolic Disorders

TAK1 plays a central role in hepatic steatosis and insulin resistance (Wang et al., 2016). Zhao et al. (2018) and Zhang Y. et al. (2019) demonstrated that deficiency of USP4 in mouse livers disrupts signaling through the TAK1/JNK-mediated insulin receptor substrate/protein kinase B/glycogen synthase kinase 3β (IRS-AKT-GSK3) axis, giving rise to non-alcoholic fatty liver disease (NAFLD). Thus, USP4 functions as a pivotal suppressor in NAFLD and related metabolic disorders.

### Immune Disease

In patients with rheumatic heart disease (RHD), an autoimmune disease, USP4 was found to be significantly elevated in CD4^+^ T cells. The deubiquitination of RORγt and IRF4 and subsequent development and differentiation of T_H_17 and T_H_2 cells mediated by USP4 might provide a possible link between USP4 and RHD (Yang et al., 2015; Guo et al., 2017).

## Cellular Regulation of USP4

**FIGURE 4:**
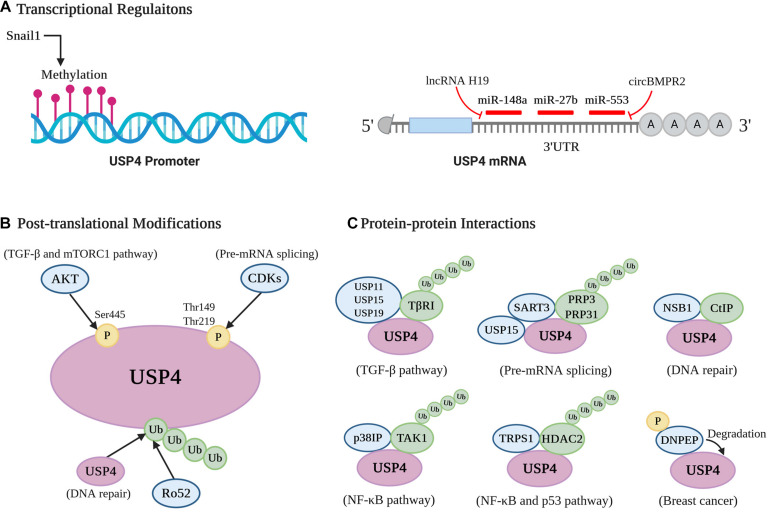
Cellular regulation of USP4. A pictorial representation of the different modes of USP4 regulation [**(A)** transcriptional regulations; **(B)** posttranslational modifications; and **(C)** protein–protein interactions].

As USP4 has a multifaceted impact on a series of physiological and pathological contexts, its stability, catalysis activity, and substrates affinity are under tight regulation. It can be organized through transcriptional regulations, PTMs, and protein–protein interactions (Figure 4).

### Transcriptional Regulations

MiR-148a and miR-27b were reported to destabilize USP4 mRNA by targeting its 3′-UTR in HCC, breast cancer, and liver fibrosis (Heo et al., 2014; Zhang et al., 2017; Wu et al., 2019). Specifically, in liver fibrosis, lower levels of miR-148a and miR-27b lead to the overexpression of USP4 in hepatic stellate cells and hepatocytes. This occurs via upregulation of long non-coding RNA H19, which acts as an miRNA sponge for miR-148a and downregulations of liver X receptor-α and cannabinoid receptor 2, respectively (Wu et al., 2019; Zhu et al., 2019). USP4 is also negatively regulated by miR-553 in breast cancer, and this inhibition could be reversed by circBMPR2, which acts as a sponge to prevent miR-553 associating with USP4 mRNA (Liang et al., 2019). DNA methylation is another transcriptional regulation of USP4. For example, when lung cancer cells interact with macrophages, Snail1 is induced and subsequently silences USP4 by promoter methylation, contributing to the inflammation and stemness associated with macrophage-promoted tumor progression (Lai et al., 2020). Furthermore, Zhong et al. (2018) indicated that hypermethylation and DNA shallow deletion are two possible mechanisms for the decreased USP4 seen in patients with lung adenocarcinoma. Thus, transcriptional mechanisms are important in regulating USP4 and in several pathological situations appear to be involved in silencing its expression.

### Posttranslational Modifications

The most prominent PTMs linked with USP4 regulation so far are phosphorylation and ubiquitination (Figure 4B). The enzyme is phosphorylated at Ser445, Thr149, and Thr219 (Zhang et al., 2012; Das et al., 2019; Deng et al., 2019). AKT directly phosphorylates USP4 at Ser445, relocating nuclear USP4 to the cytoplasm and membrane with the help of 14-3-3 protein, as well as enhancing its stability and deubiquitinating activity. Notably, this modification is essential for the USP4-mediated deubiquitination of TβRI and Rheb (Zhang et al., 2012; Deng et al., 2019). Moreover, the stabilization of TβRI and subsequent activation of AKT positively feedback to AKT’s affinity and phosphorylation of USP4. Phosphorylation of USP4 at the alternate residues, Thr149 and Thr219, is mediated by cyclin-dependent kinases and exerts a negative role in pre-mRNA splicing through blocking USP4 interactions with squamous cell carcinoma antigen recognized by T cells 3 (SART3) (Das et al., 2019).

USP4 also possesses various ubiquitination sites, including the zinc-binding motif Cys residues Cys-461, Cys-464, Cys-799, and Cys-802 (Liu et al., 2015; Wijnhoven et al., 2015). USP4 is ubiquitinated by the ubiquitin ligase Ro52, whose deubiquitination, in turn, is facilitated by USP4 (Wada and Kamitani, 2006; Wada et al., 2006). Besides, the ubiquitination level of USP4 is determined by its catalytic activity. Expression studies using an enzyme-dead mutant of USP4 demonstrated that ubiquitination levels were increased compared to wild-type USP4 (Wijnhoven et al., 2015). Of note, USP4 can deubiquitinate itself, and this autodeubiquitination function is crucial for the USP4-CtIP interaction, which occurs in HR (Liu et al., 2015; Wijnhoven et al., 2015). It seems that phosphorylation and ubiquitination are involved in USP4-mediated deubiquitinating processes by acting as a positive or negative regulator. More importantly, the status of these two PTMs may be antagonistic. Zhang et al. (2012) demonstrated that abolishing AKT-mediated phosphorylation increases the level of USP4 conjugation with polyubiquitin chains, consequently impairing enzyme stability and activity.

### Protein–Protein Interactions

Several proteins have been identified to mediate the stability and/or activity of USP4 by directly interacting with it (Figure 4C). Foremost, USP4 can bind to itself, whereas other DUBs such as USP11, USP15, and USP19 were observed as binding partners of USP4 that assist in deubiquitinating TβRI (Zhang et al., 2012). During the pre-mRNA splicing process, the U4/U6 recycling protein SART3 functions as a substrate targeting factor of USP4 (Song et al., 2010; Park et al., 2016; Kwon et al., 2017). By binding to the linker between the DUSP and UBL domain of USP4, SART3 can shuttle USP4 to the nucleus from the cytosol through its NLS. The USP4^sart3^ complex helps to recruit the substrate PRP3, as well as increase the deubiquitinating activity of USP4 (Song et al., 2010; Park et al., 2016). Moreover, as a substrate recruiting factor of USP15 either (Long et al., 2014; Zhang et al., 2016), SART3 can simultaneously bind USP4 and USP15, serving as a platform to deubiquitinate PRP31 and PRP3 (Das et al., 2017). Another protein, NBS1, which is a part of the MRN complex, interacts with the DUSP domain of USP4 and recruits the enzyme to DNA damage sites where it colocalizes with CtIP (Liu et al., 2015).

p38IP was found to be a necessary regulator in USP4-mediated deubiquitination of TAK1. It can specifically bind to the fingers subdomain of USP4 through a cavity-like structure and serves to recruit USP4 to the TAK1 complex (Wang X. D. et al., 2020). This recruitment is dependent on p38IP with a higher affinity to ubiquitinated TAK1 upon TCR, LPS, or TNF-α stimulation (Fan et al., 2011; Wang X. D. et al., 2020). Similarly, trichorhinophalangeal syndrome type I (TRPS1) functions as a scaffold protein involved in the USP4-induced stabilization of HDAC2 through bringing them together to form the TRPS1-USP4-HDAC2 complex (Wang et al., 2018). Finally, USP4 was reported to interact with and be degraded by aspartyl aminopeptidase (DNPEP), a metalloenzyme belonging to the M18 aminopeptidase family, resulting in suppression of its oncogenic role in breast cancer. Moreover, phosphorylation of DNPEP by p21-activated kinase 5 (PAK5) is indispensable in this process (Geng et al., 2020).

## Therapeutic Developments for Targeting USP4

Research carried out in the past few decades has uncovered the essential roles of DUBs in disease, especially in cancer, giving sufficient evidence to consider these molecules as potential pharmaceutical targets. USP4 is no exception, but progress in the development of USP4 inhibitors remains slow, with only two compounds reported so far (Okada et al., 2013; Nguyen et al., 2019). Xie et al. (2005) isolated a small compound from Chinese mushroom *Thelephora vialis*, namely, vialinin A. Vialinin A was initially discovered to exhibit an effective anti-inflammatory activity, but later it was identified as a micromolar inhibitor of USP4 (IC_50_ = 1.5 μM), USP5/isopeptidase T (IC_50_ = 5.9 μM), and UCH-L1 (IC_50_ = 22.3 μM) (Ye et al., 2007; Okada et al., 2013). Treatment with vialinin A not only inhibits colorectal tumor growth but also interferes with USP4-mediated immune responses and pathological scarring (Yang et al., 2015; Guo et al., 2017; Lin et al., 2017; Deng et al., 2019; Zhang J. et al., 2019). NR, a eurhodin dye–containing aminophenazine structure, was recognized as another non-competitive DUB inhibitor with selectivity for USP4 (IC_50_ = 50 μM). The tumor-promoting role of USP4 in colorectal cancer can also be reversed by NR through decreasing β-catenin stability (Nguyen et al., 2019). These findings suggest that targeting USP4 holds promise as cancer therapy, but highly specific nanomolar-range drugs still need to be discovered and developed.

## Conclusion

This review illustrates the current literature on USP4 that sheds light on the prominent and multifaceted role of USP4 in normal and pathological states. USP4 deubiquitinates a wide range of protein substrates under strict and diverse regulatory conditions. Major cancer pathways including p53, Wnt/β-catenin, TGF-β, and NF-κB signaling pathways are modulated by USP4, whereas regulation of TGF-β and NF-κB signaling pathways is also involved in other diseases, including spinal cord injury, liver fibrosis and cirrhosis, and pathological scarring (Xiao et al., 2012; Zhang et al., 2012; Yun et al., 2015; Cao et al., 2016; Hwang et al., 2016; Jiang et al., 2017; Qiu et al., 2018; Qin et al., 2019; Wu et al., 2019; Huang et al., 2020; Lai et al., 2020). Together, these studies provide both insights into USP4 biology as well as its applicability as a potential treatment target for many diseases.

Specifically, in some diseases such as hepatic I/R injury, USP4 is upregulated to exert a protective function (Zhou J. et al., 2019). Moreover, USP4 and its paralog USP15 play both promoting and suppressing roles in cancer (Chou et al., 2017). This highlights the need to carefully evaluate the role of USP4 in individual diseases. Indeed, the present understanding of USP4 is insufficient, and there are some contradictory findings that remain to be explained. For example, USP4 removes K48-linked ubiquitin chains from TRAF6 in cells infected by EV71 (Xu et al., 2018), whereas in TNF-α–induced inflammation, TRAF6 K63-linked polyubiquitinated chains are cleaved (Xiao et al., 2012). Hence, USP4 exerts opposing functions in the activation of the NF-κB signaling pathway. In breast cancer, Wang et al. (2018) reported that USP4 facilitates cell proliferation through promoting the stability of HDAC2, whereas Li Y. et al. (2016) found it inhibits cell growth by deubiquitinating PCD4.

Deciphering the mechanisms determining USP4 specificity toward different types of ubiquitin chains and affinity with different substrates is a far-reaching task that will likely contribute to explaining the multidimensional roles of USP4. As substrates may compete for USP4 binding occurring with USP7, this must also be factored into this analysis. For example, the competition between the tumor suppressor p53 and its E3 ubiquitin ligase HDM2 serves as a switch to fine-tune the cellular level of p53. This is due to a common mode of interaction found in USP7 that all the substrates recognized by the TRAF-like domain share a common P/AxxS motif or ExxS motif (Pozhidaeva and Bezsonova, 2019). Therefore, revealing the interaction modes of USP4 seems to be valuable. USP4 is also subject to diverse regulation within the cell with transcriptional mechanisms being involved in determining USP4 levels, whereas phosphorylation, ubiquitination, and binding partners of USP4 modulate USP4 catalytic activity, subcellular localization, and association with substrate proteins. Different regulations of USP4 may result in the different roles of USP4 in cells or situations. Thus, not only the downstream substrates but also the upstream regulators of USP4 need to be further explored.

Finally, to comment on the slow progress made in developing USP4 as a treatment target. The two inhibitors showing preclinical activity are not USP4-specific and have never been considered for use in clinical trials. Even if suitable inhibitors became available, the use of USP4 as a therapeutic target must be considered carefully as it plays a tumor-suppressing role in some types of cancer. Thus, in addition to identifying more effective USP4 inhibitors targeting USP4, future research should also focus on understanding which cancers would likely benefit from this treatment along with other diseases where USP4 would inhibit progression.

## Author Contributions

BH drafted the manuscript. DZ, KZ, and YW drew the pictures. LP and QF revised the manuscript. XM checked and modified the manuscript. All authors contributed to the article and approved the submitted version.

## Conflict of Interest

The authors declare that the research was conducted in the absence of any commercial or financial relationships that could be construed as a potential conflict of interest.
